# Quantitative comparison of wideband low-latency phase-locked loop circuit designs for high-speed frequency modulation atomic force microscopy

**DOI:** 10.3762/bjnano.9.176

**Published:** 2018-06-21

**Authors:** Kazuki Miyata, Takeshi Fukuma

**Affiliations:** 1 Nano Life Science Institute (WPI-NanoLSI), Kanazawa University, Kakuma-machi, Kanazawa 920-1192, Japan; 2 Division of Electrical Engineering and Computer Science, Kanazawa University, Kakuma-machi, Kanazawa 920-1192, Japan

**Keywords:** calcite dissolution process, frequency modulation atomic force microscopy, high-speed atomic-resolution imaging, phase-locked loop

## Abstract

A phase-locked loop (PLL) circuit is the central component of frequency modulation atomic force microscopy (FM-AFM). However, its response speed is often insufficient, and limits the FM-AFM imaging speed. To overcome this issue, we propose a PLL design that enables high-speed FM-AFM. We discuss the main problems with the conventional PLL design and their possible solutions. In the conventional design, a low-pass filter with relatively high latency is used in the phase feedback loop, leading to a slow response of the PLL. In the proposed design, a phase detector with a low-latency high-pass filter is located outside the phase feedback loop, while a subtraction-based phase comparator with negligible latency is located inside the loop. This design minimizes the latency within the phase feedback loop and significantly improves the PLL response speed. In addition, we implemented PLLs with the conventional and proposed designs in the same field programmable gate array chip and quantitatively compared their performances. The results demonstrate that the performance of the proposed PLL is superior to that of the conventional PLL: 165 kHz bandwidth and 3.2 μs latency in water. Using this setup, we performed FM-AFM imaging of calcite dissolution in water at 0.5 s/frame with true atomic resolution. The high-speed and high-resolution imaging capabilities of the proposed design will enable a wide range of studies to be conducted on various atomic-scale dynamic phenomena at solid–liquid interfaces.

## Introduction

Frequency modulation atomic force microscopy (FM-AFM) is a powerful tool for investigating atomic- and molecular-scale structures of sample surfaces in various environments [1]. It has been used under ultrahigh vacuum conditions for high-resolution imaging of various materials, including metals, semiconductors, metal oxides, and organic molecules [2–5]. Furthermore, recent advances in FM-AFM have enabled atom manipulation and identification at room temperature [6–7], as well as atomic-scale imaging of intramolecular structures at low temperatures [8]. The liquid-environment applications of FM-AFM have also been intensively explored. So far, this method has been utilized for atomic-resolution imaging of inorganic crystals [9–14] and subnanometer-scale imaging of biomolecules [15–17]. Moreover, three-dimensional (3D) imaging techniques have been developed based on FM-AFM and used to visualize 3D distributions of hydration structures as well as flexible surface structures at solid–liquid interfaces [18–22].

Although FM-AFM enables atomic-scale measurements to be performed in vacuum and in liquids, its imaging speed is relatively slow (ca. 1 min/frame). High-speed amplitude modulation AFM has been developed [23] and employed to visualize the dynamic behavior of biomolecules in liquids at 10–100 ms/frame [24]. However, its spatial resolution is limited to the nanometer scale and it can only be employed in liquid environments. Thus, even with advanced AFM techniques, it has been difficult to visualize atomic-scale processes occurring at time scales less than about 1 min. This shortcoming has hindered investigation of the atomistic mechanisms of various interfacial phenomena, including thin-film formation, crystal growth and dissolution, metal corrosion, and catalytic reactions in vacuum and in liquids.

This problem could be solved by enhancing the FM-AFM operation speed. The development of high-speed FM-AFM requires improvements in bandwidth or resonance frequency of all of the components constituting the tip–sample distance regulation loop, such as the cantilever, cantilever excitation unit, cantilever deflection sensor, scanner, feedback controller, and phase-locked loop (PLL) circuit. In particular, the PLL circuit is often a primary speed-limiting factor in FM-AFM systems. Thus, the development of a high-speed PLL circuit is the key to improve the FM-AFM operation speed.

In a typical PLL circuit for FM-AFM, a multiplication-based phase comparator (PC) is used to detect the phase difference between an input signal and a reference signal. In a multiplication-based PC, a low-pass filter (LPF) is employed to eliminate higher harmonics generated by the multiplication of an input signal and a reference signal. However, a LPF has a relatively high latency (i.e., long time delay) at its pass band, often limiting the PLL circuit response speed. To overcome this limitation, we recently proposed a design using a subtraction-based PC with a high-pass filter (HPF) [25]. Owing to the low latencies of HPFs at their pass bands, the proposed design enables a significant improvement of the PLL circuit response speed. With the developed high-speed PLL, we recently demonstrated atomic-resolution imaging in a liquid at 2 s/frame [26]. However, the design and principle of the developed PLL have not been reported in detail. In addition, the bandwidth and latency improvements achieved using the proposed design have not been quantitatively compared with those of the other PLL designs.

In this report, we present detailed comparisons of different PLL designs and thereby clarify the design concept of the developed high-speed PLL circuit. Furthermore, we implemented a conventional PLL circuit as well as the proposed PLL circuit in the same digital signal-processing platform to conduct quantitative and direct performance comparisons. We also present the result of high-speed FM-AFM imaging of calcite dissolution with atomic-scale resolution to demonstrate the applicability of the proposed PLL design to practical experiments.

## Experimental

### Digital circuits implementation

All of the digital circuits tested in this study were implemented in the same field programmable gate array (FPGA) chip (Vertex-5, Xilinx). The FPGA clock frequency and the analog-to-digital converter (ADC) and digital-to-analog converter (DAC) sampling rates were set to 100 MHz and 100 MSPS, respectively. The design parameters of the digital filters were objectively determined using the following design guidelines to achieve fair comparison. The LPF and HPF were implemented as Butterworth filters. The gain at the stop band was restricted to less than −40 dB while the amplitude of the gain ripple at the pass band was held less than 0.1 dB. Taking into account the latency and resource, a second-order LPF, second-order HPF, fourth-order band-pass filter (BPF), and the 10th-order band-elimination filter (BEF) were used (see Figure 2(iii) below for the group delays of these filters).

### PLL performance measurements

The amplitude and phase curves of the implemented PLLs were measured using the commercially available frequency response analyser (FRA5097, NF). The waveforms depicted below in Figure 6 were obtained using the commercially available oscilloscope (TDS2002B, Tektronix). The experimental data presented below in Figure 7 and Figure 8 were obtained using a standard-size cantilever (NCH, Nanoworld; *f*_0_ = 151.46 kHz, *k* = 41.3 N/m and *Q* = 9) and an ultra-short cantilever (USC, Nanoworld; *f*_0_ = 3.44 MHz, *k* = 59.9 N/m and *Q* = 7). The cantilever vibrations were excited and detected using a highly stable custom-built photothermal excitation system and low-noise optical beam deflection sensor, respectively [27–30]. The noise spectra shown below in Figure 8 were obtained using the commercially available AFM controller (ARC2, Asylum Research).

### Sample preparation and imaging conditions

A 5 × 5 × 2 mm^3^ calcite crystal (Crystal Base Co., Ltd.) was used for the FM-AFM experiment. The sample was glued onto the sample holder. Immediately after cleavage of the sample with a razor blade, 50 μL of Milli-Q water was deposited onto the sample surface. Then, high-speed FM-AFM imaging was performed in the deposited water using an AC55 (Olympus) cantilever (*f*_0_ = 1.53 MHz, Δ*f* = 1.6 kHz, *A* = 0.1 nm). The cantilever vibrations were excited and detected using the same setups that were employed for the PLL performance measurements. For the high-speed operation of the FM-AFM system, we used a custom-built high-speed scanner and AFM controller [25,31].

## Results and Discussion

### Basic PLL circuit design

#### PLL circuit with multiplication-based PC

**Figure 1:**
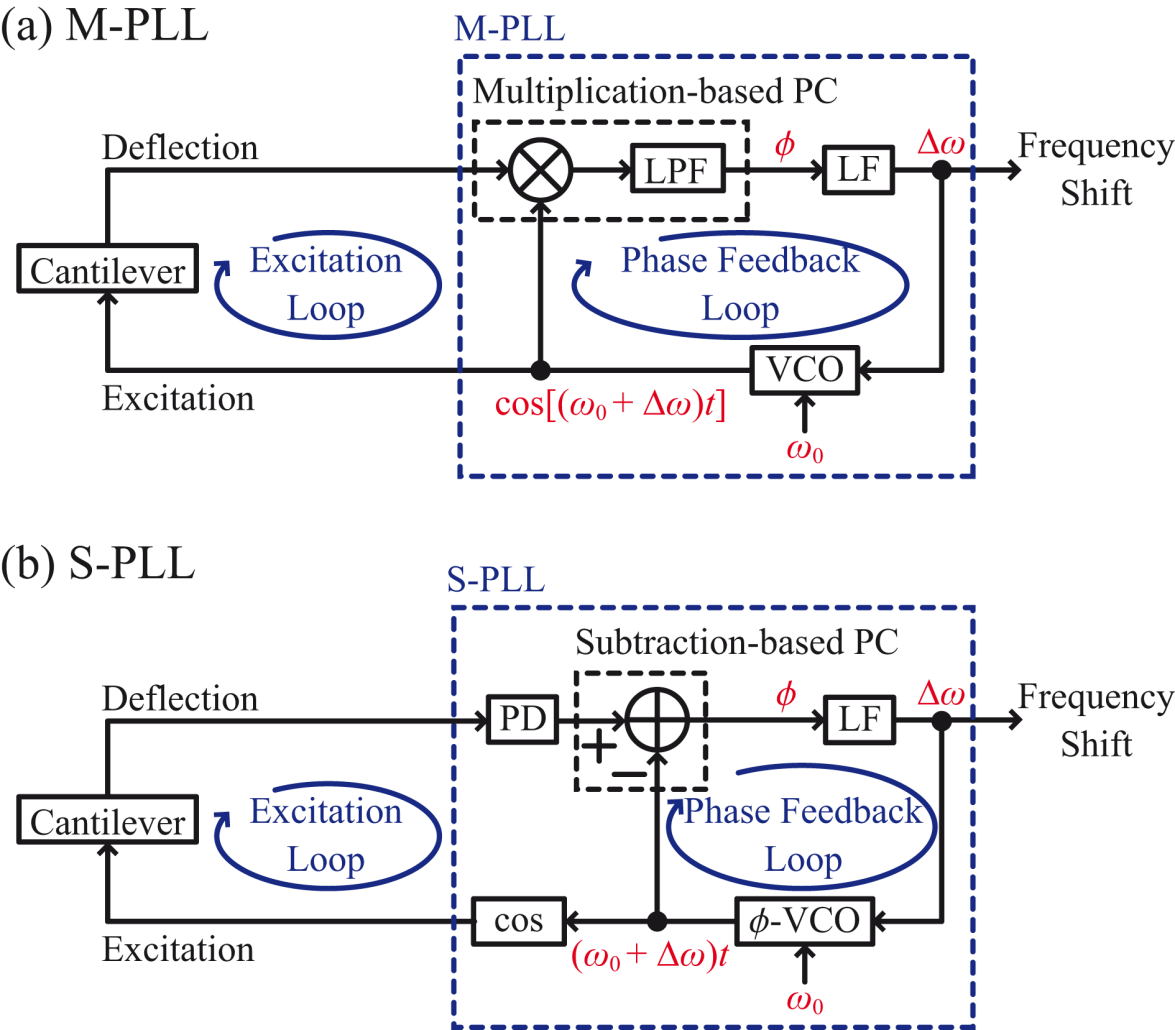
Basic designs of (a) M-PLL and (b) S-PLL circuits.

Figure 1a depicts the basic design of a typical PLL circuit using a multiplication-based PC (M-PLL) for FM-AFM. In this design, a voltage-controlled oscillator (VCO) is utilized to generate a sine wave, cos[(ω_0_ + Δω)*t*], which is referred to as a reference signal. The VCO output frequency changes in proportion to the VCO input signal around a fixed free-running frequency (i.e., the cantilever resonance frequency, ω_0_). In a PLL circuit, the phase difference 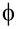 between the reference and PLL input signal (i.e., cantilever deflection signal) is detected. The detected signal is fed into a loop filter (LF). This LF effectively works as a proportional-integral feedback controller. Specifically, the LF controls the VCO input signal so that 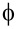 remains constant. In this way, the VCO, PC, and LF form a phase feedback loop, as illustrated in Figure 1. In a steady state, the frequency of the VCO output agrees with that of the PLL input. Thus, the VCO input changes in proportion to the frequency shift Δω of the PLL input. This signal is output from the PLL and used for tip–sample distance feedback control. Meanwhile, the VCO output is employed to excite cantilever oscillations, forming the cantilever excitation loop depicted in Figure 1a.

**Figure 2:**
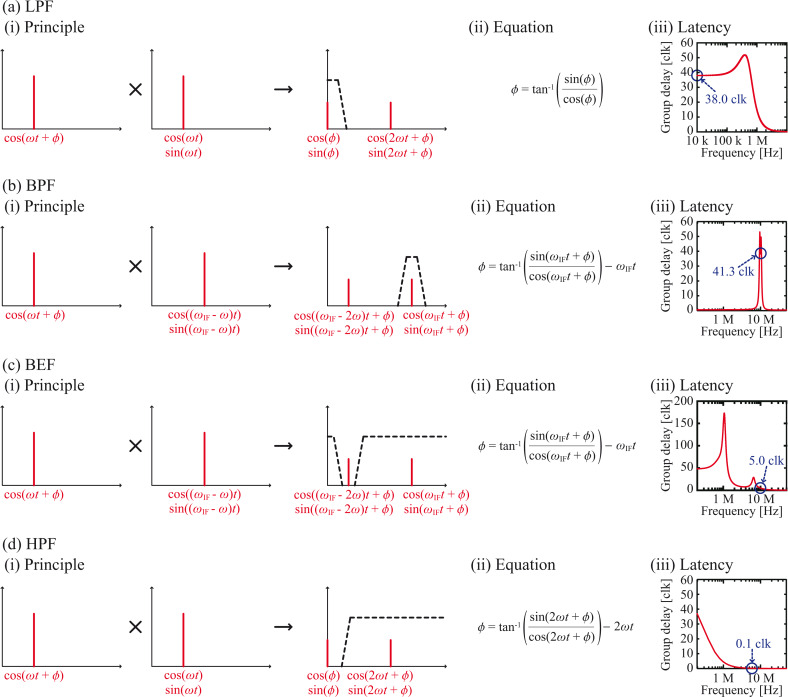
Basic concepts of different PC designs using (a) a LPF, (b) a BPF, (c) a BEF and (d) a HPF. (i) Principles. (ii) Equations for calculating 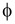. (iii) Examples of latency-versus-frequency curves. The design parameters were individually optimized for a 3 MHz input signal and 100 MHz clock frequency for digital signal processing.

Similarly to a general lock-in amplifier, an M-PLL utilizes a multiplication-based PC. Figure 2a shows the basic principle of a multiplication-based PC with a LPF. In an M-PLL, two reference signals, cos[(ω_0_ + Δω)*t*] and sin[(ω_0_ + Δω)*t*], generated by a VCO are individually multiplied by a PLL input signal, cos[(ω_0_ + Δω)*t* + 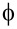], where 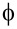 denotes the phase difference between the PLL input and reference signal, producing dc and 2ω components (Figure 2a(i)). The 2ω components are rejected by the LPF, while the dc components are used to calculate 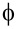 with a Cartesian-to-polar coordinate converter (Figure 2a(ii)). However, a LPF has a relatively high latency at its pass band, severely decreasing the response speeds of multiplication-based PCs. In addition, when a LPF is used in a PLL circuit, it is placed in the phase feedback loop, so its influence is further amplified.

For quantitative analysis, we present the latency-versus-frequency curves of different filters in Figure 2(iii). The design parameters were individually optimized for a 3 MHz input signal and 100 MHz clock frequency for digital signal processing. As explained in the Experimental section in detail, we implemented these parameters based on objective criteria to achieve fair comparison. For a LPF (Figure 2a(iii)), the latency is 38 clocks (380 ns), which is large enough to impact the PLL response time significantly.

#### PLL circuit with subtraction-based PC

Recently, we proposed a PLL circuit using a subtraction-based PC (S-PLL) [32]. Figure 1b depicts the basic design of an S-PLL circuit. In this design, a phase-output VCO (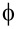-VCO) is utilized to produce the phase (ω_0_ + Δω)*t* of the reference signals. Similarly to the output frequency of a VCO, that of the 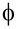-VCO changes in proportion to its input signal around ω_0_. A PLL input signal is fed into a phase detector (PD) that outputs the phase of the input signal, (ω_0_ + Δω)*t* + 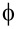. The detected phase signal is compared with the 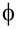-VCO output by subtraction to obtain 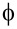. The latency of the subtractor is negligible, so no components in the phase feedback loop have high latencies. Thus, the response of an S-PLL is much faster than that of an M-PLL.

In the S-PLL design we first proposed previously, a Hilbert transformer was utilized as a PD [32]. Although the developed S-PLL exhibited excellent performance under ideal operating conditions, it was not practically useful in an AFM experiment. For FM-AFM, cantilevers with a wide *f*_0_ range (100 kHz–4 MHz) are used. However, a Hilbert transformer with such a wide input range has a high latency at its pass band. This high latency results in a slow cantilever excitation loop response time. Therefore, in spite of the fast response of the phase feedback loop, the investigated S-PLL circuit was not suitable for high-speed FM-AFM operation.

One of the most common methods of expanding the input frequency range is heterodyne conversion using a BPF (Figure 2b). In this method, an input signal with a frequency of ω is multiplied by sine and cosine waves with frequencies of (ω_IF_ − ω), producing ω_IF_ and (ω_IF_ − 2ω) components (Figure 2b(i)). While the (ω_IF_ − 2ω) component is rejected by the BPF, the ω_IF_ component is routed to a Cartesian-to-polar coordinate converter, where its phase (ω_IF_*t* + 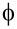) is detected (Figure 2b(ii)). The free-running frequency of the 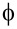-VCO is set to ω_IF_ so that it outputs a signal with a phase of ω_IF_*t*. By comparing the phase of the two signals using a subtraction-based PC, 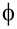 can be obtained. However, a BPF has a relatively high latency at its pass band, which limits the excitation loop response speed. In the example shown in Figure 2b(iii), the latency of the BPF is 41.3 clocks (413 ns) at its pass band. This high latency prevents its application in high-speed FM-AFM.

To solve this problem, a BEF can be employed instead of a BPF (Figure 2c). In this design, only the (ω_IF_ − 2ω) component is rejected by the BEF, which is unlike the design with a BPF, in which only the ω_IF_ component is passed to the following stage (Figure 2b). The latency of a BEF at its pass band is much lower than that of a BPF. Thus, it can greatly improve the excitation loop response speed. In the example shown in Figure 2c(iii), the latency of the BEF at its pass band is 5 clocks (50 ns), which is much lower than that of a BPF. However, this design has another practical issue. When a cantilever with a relatively low resonance frequency is used, the frequency difference between the two components generated by multiplication (i.e., (ω_IF_ − 2ω) and ω_IF_) becomes smaller. Although the (ω_IF_ − 2ω) component can be eliminated with a high-*Q* BEF, this filter causes significant delay of the filtered signal at ω_IF_. Thus, this design can only be applied practically in experiments with cantilevers of high resonance frequency.

The BEF problem becomes more serious when the ratio between the frequencies of the signals to be passed and rejected approaches 1. In this manuscript, this ratio is defined as *R* = ω_IF_/(ω_IF_ − 2ω). To minimize the BEF problem, *R* should be maximized by adjusting ω_IF_. According to the definition, *R* is maximized when ω_IF_ equals 2ω. When this condition is satisfied, unwanted components always appear around dc. Thus, a HPF instead of a BEF can be employed to eliminate them, as shown in Figure 2d. By maximizing *R*, this design provides the lowest latency for a given cantilever resonance frequency. In the example depicted in Figure 2d(iii), the latency at the pass band is 0.1 clock (1 ns) and is therefore negligible. This design enabled us to develop a wideband and low-latency PLL circuit with a wide input frequency range.

### Performance comparison

#### PLL implementation

**Figure 3:**
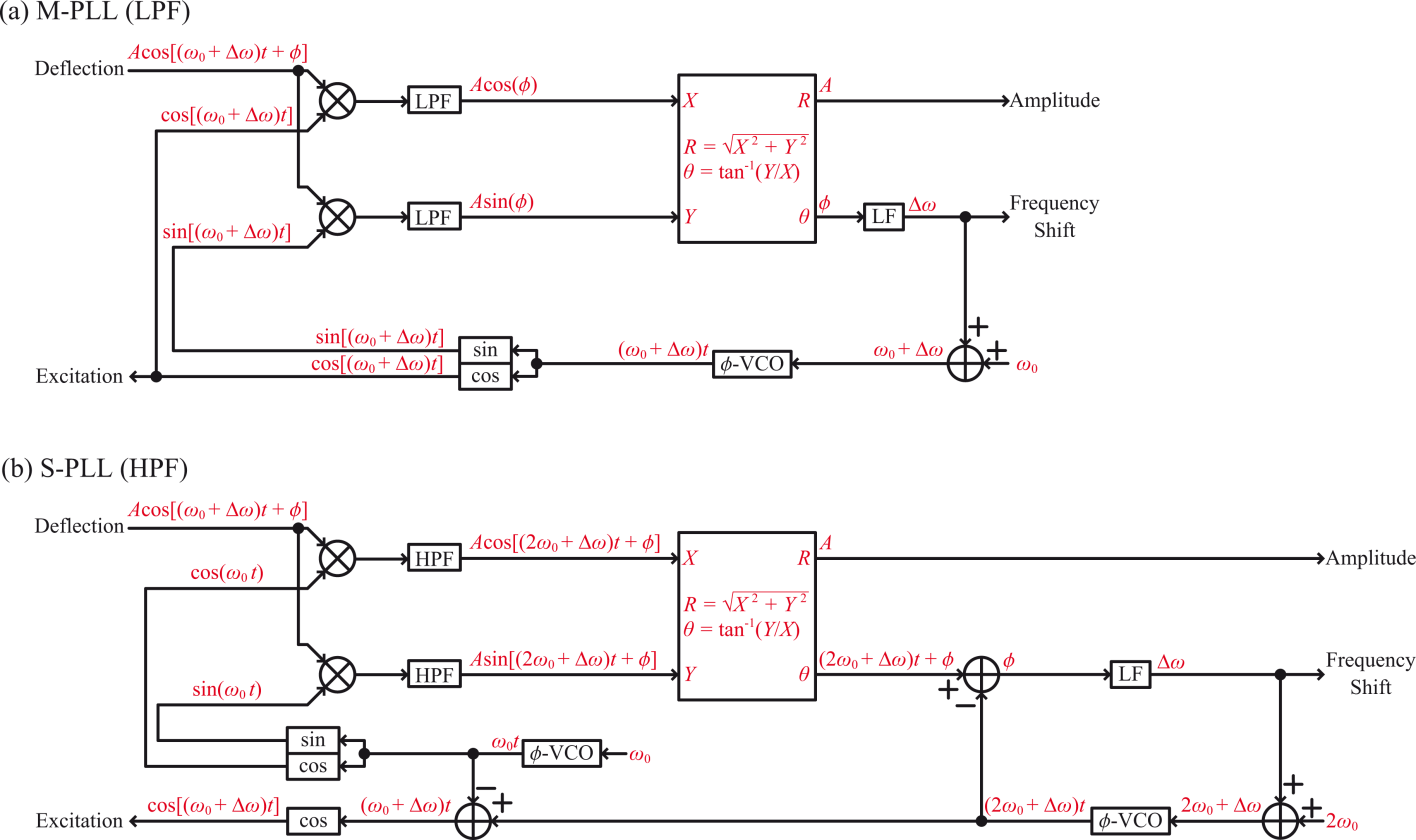
Block diagrams of the implemented (a) M-PLL and (b) S-PLL.

In this study, we implemented the M-PLL with a LPF and the S-PLL with a HPF on the same FPGA chip to compare their performances quantitatively. Figure 3 provides the block diagrams of the implemented PLLs, where the FPGA clock frequency and the ADC and DAC sampling rates were set to 100 MHz and 100 MSPS, respectively. The CORDIC algorithm was utilized to calculate the amplitude *R* = (*X*^2^ + *Y*^2^)^1/2^ and phase θ = tan^−1^(*Y*/*X*) signals from the *XY* inputs. As described in the Experimental section in detail, the parameters used to implement the two PLLs were objectively determined by employing the same design guidelines for fair comparison. Hereafter, we refer to the two designs as the LPF and HPF designs.

#### PD–PC units

**Figure 4:**
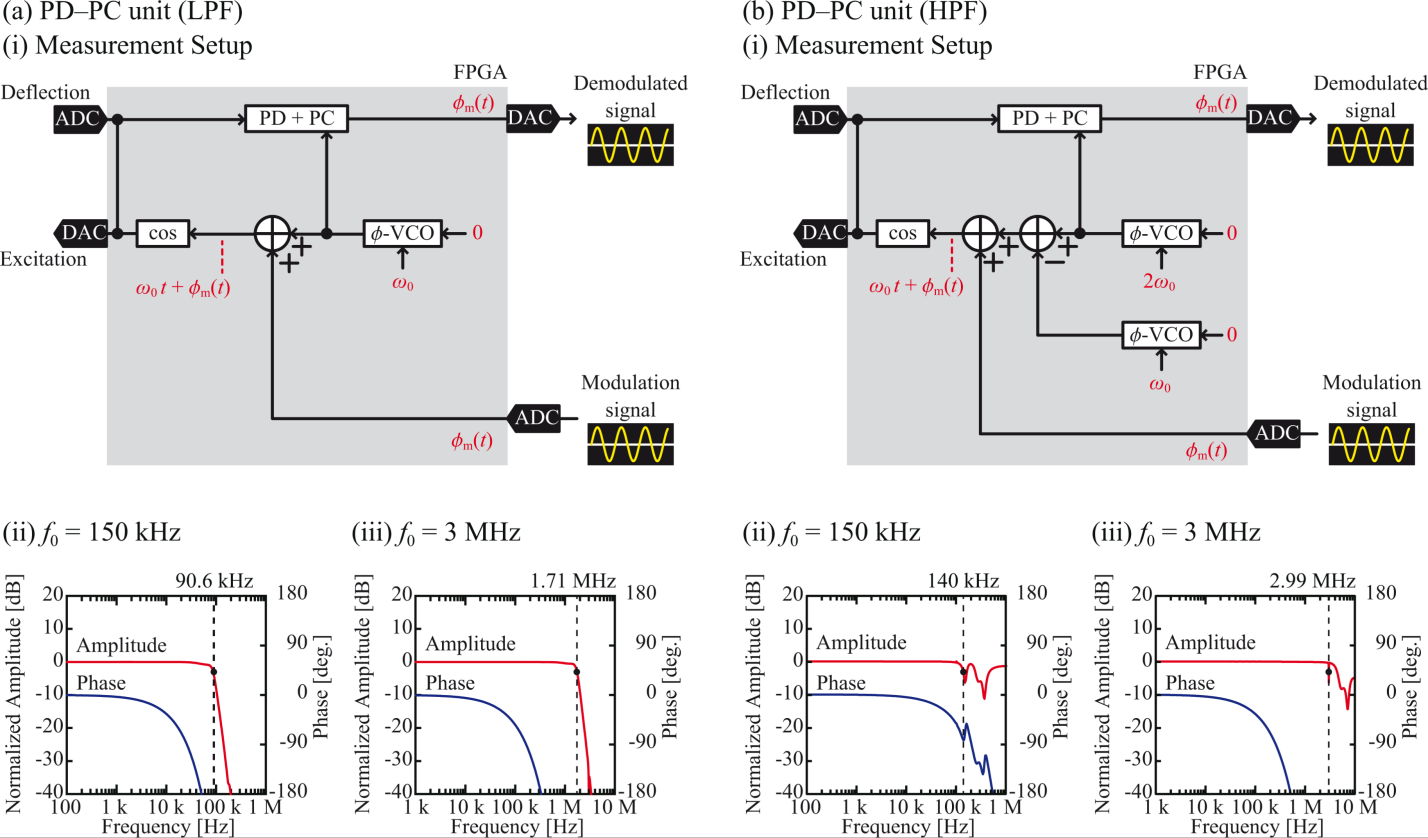
Performance of the implemented PD–PC units with (a) a LPF and (b) a HPF. (i) Block diagram of the setup. Frequency responses of the PD–PC units with (ii) *f*_0_ = 150 kHz and (iii) *f*_0_ = 3 MHz.

Figure 4(i) depicts the experimental setups used to measure the frequency responses of the implemented PD–PC units. In these setups, a sine wave 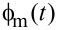 (= cos ω_m_*t*) generated by an external oscillator is fed into the FPGA through the ADC. This signal is added to the 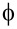-VCO output (ω_0_*t*) and fed into the cosine converter to generate the phase-modulated cantilever excitation signal, cos[ω_0_*t* + 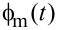]. The generated excitation signal is not output from the DAC, but directly fed into the PD in the FPGA. Thus, the excitation signal also works as a false cantilever deflection signal and is demodulated by the PD–PC unit to estimate the latency of the PD–PC unit itself by excluding the influences of the other components in the cantilever excitation loop such as the ADC/DAC, cantilever, cantilever excitation unit, and cantilever deflection sensor. The demodulated signal 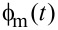 is output from the DAC and analyzed by an external frequency response analyzer. We used *f*_0_ values of 150 kHz and 3 MHz, which are typical resonance frequencies of the standard-size cantilever (NCH, Nanoworld) and the ultra-short cantilever (USC, Nanoworld) in water, respectively.

Figures 4(ii) and 4(iii) present the frequency responses of the PD–PC units. From the amplitude curves, the bandwidths defined as the frequencies corresponding to −3 dB attenuation were estimated and are presented in Table 1 and Table 2. The amplitude curve obtained with the LPF design exhibits a simple response, where the bandwidth is limited by the cut-off frequency of the LPF. Meanwhile, the curve obtained with the HPF design displays sharp negative peaks at ω_0_ and 2ω_0_ due to the sideband signal rejections by the HPF. Even in this case, applying the same definition of the bandwidth as the negative peak at ω_0_ will practically limit the available frequency range in actual experiments.

**Table 1:** Bandwidths and latencies of the developed PD–PC unit, PLL, and PLL with a cantilever (*f*_0_ = 150 kHz, NCH, Nanoworld).

	PD–PC		PLL		PLL with cantilever	
			
	bandwidth [kHz]	latency [μs]	bandwidth [kHz]	latency [μs]	bandwidth [kHz]	latency [μs]

M-PLL (LPF)	90.6	9.1	15.4	22.8	2.55	66.0
S-PLL (HPF)	140	1.1	75.0	6.6	4.21	44.8
improvement	×1.54	×0.12	×4.87	×0.29	×1.65	×0.68

**Table 2:** Bandwidths and latencies of the developed PD–PC unit, PLL, and PLL with a cantilever (*f*_0_ = 3 MHz, USC, Nanoworld).

	PD–PC		PLL		PLL with cantilever	
			
	bandwidth [kHz]	latency [μs]	bandwidth [kHz]	latency [μs]	bandwidth [kHz]	latency [μs]

M-PLL (LPF)	1710	1.5	62.6	4.1	56.2	4.6
S-PLL (HPF)	2990	1.0	305	1.6	165	3.2
improvement	×1.75	×0.65	×4.87	×0.39	×2.94	×0.70

In Figure 4, the phase curves appear to be non-linear since the phase is plotted against the logarithm of the frequency. However, if the phase is plotted against the linear frequency, the plots appear to be almost perfectly linear. These results suggest that the phase delay 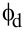 is caused by the constant latency *τ*_d_, having no frequency dependence and being described by the following equation:

[1]
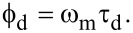


Thus, we fitted the phase curves using this equation and estimated *τ*_d_, which is given in Table 1 and Table 2. The latency is mainly caused by the ADC/DAC and digital signal processing in the FPGA. Since the former should be constant, the difference originates from the signal processing algorithm and ω_0_. More specifically, the signal processing latency is mostly caused by the filter (e.g., LPF or HPF) due to the absence of phase feedback loops in the tested systems.

Table 1 and Table 2 demonstrate that the HPF design provides a wider bandwidth and a lower latency than the LPF design. The bandwidth achieved using the HPF design is nearly *f*_0_, while that yielded by the LPF design is approximately (2/3)*f*_0_. In the HPF design, the 2*f*_0_ signal generated by multiplication is not attenuated by the HPF except in the narrow frequency range near *f*_0_ and 2*f*_0_. In contrast, in the LPF design, the amplitude response becomes gradually attenuated as *f*_m_ increases and approaches the LPF cut-off frequency. Thus, the LPF design generally has a narrower bandwidth than the HPF design. For the latency, Table 1 and Table 2 reveal that the difference between the two designs increases with decreasing *f*_0_. The latency of the HPF at its pass band is negligible regardless of *f*_0_, while that of the LPF increases with decreasing *f*_0_. These characteristics explain the difference between the *f*_0_ dependences of the two designs.

#### PLL

**Figure 5:**
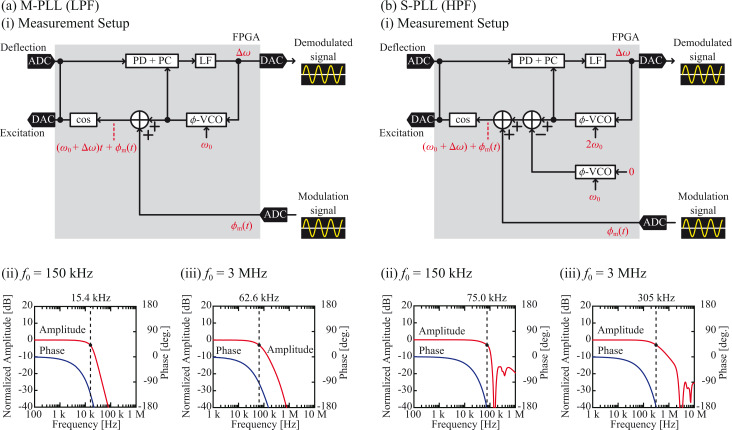
Performances of the (a) M-PLL with the LPF and (b) S-PLL with the HPF. (i) Block diagrams of the measurement setups. Frequency responses of the PLLs with (ii) *f*_0_ = 150 kHz and (iii) *f*_0_ = 3 MHz.

Figure 5(i) depicts the setups utilized to measure the bandwidth and latency of the implemented PLL. These setups are mostly the same as those in Figure 4(i), but with a LF and a phase feedback line added to each of them. Similarly to the measurements of the PD–PC units, a real cantilever was not used; instead, the cantilever excitation signal was directly fed into the PLL in the FPGA to enable measurement of the frequency response of the PLL itself. In the measurements, we modulated the phase to induce Δω. This method perfectly reproduces what happens in actual FM-AFM experiments, in which the tip–sample interaction force first induces a phase shift of the cantilever deflection signal. This phase shift is then compensated by the phase feedback loop and is converted to a frequency shift.

Figures 5(ii) and 5(iii) present the frequency responses of the M-PLL with a LPF and the S-PLL with a HPF. In both cases, the response is flat up to a certain frequency and steeply decreases at higher frequencies. The decreases are due to the limited bandwidths of the phase feedback loops. For the S-PLL, sharp negative peaks are observable at *f*_0_ and 2*f*_0_. These peaks originate from the negative peaks observable in the amplitude responses of the PD–PC units (Figure 4b). The PLL bandwidth can be enhanced by increasing the LF gain. However, if the gain is too high, the phase feedback loop becomes unstable and starts to oscillate. In this experiment, we set the gain to the maximum value in the range in which the feedback loop was stable.

**Figure 6:**
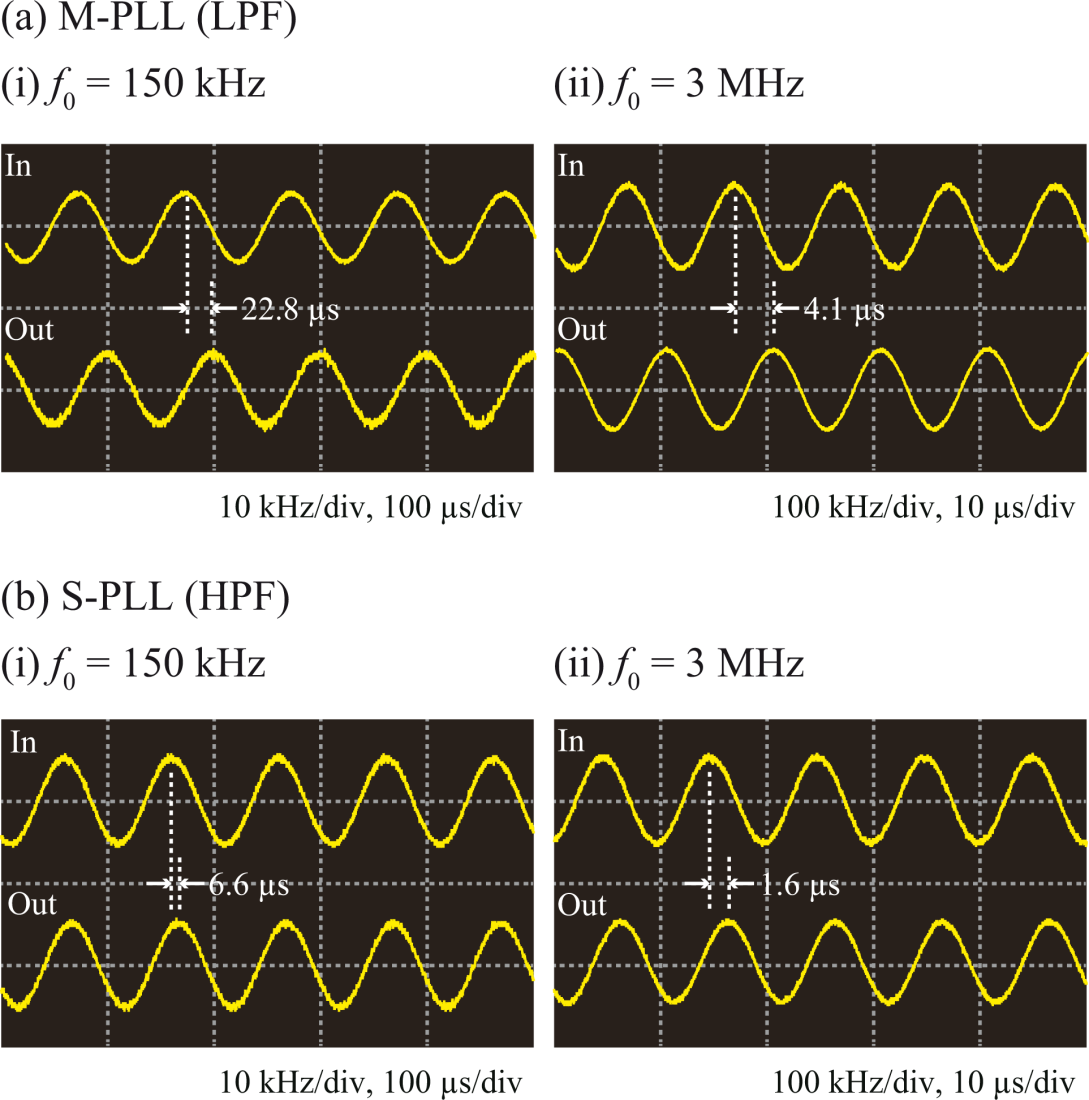
(a) M-PLL and (b) S-PLL waveforms at (i) *f*_0_ = 150 kHz and (ii) *f*_0_ = 3 MHz.

Figure 6 depicts the waveforms of the phase modulation and demodulated frequency shift signals measured after optimizing the LF gain. The modulation frequency ω_m_ was set to 10 kHz and 100 kHz for *f*_0_ values of 150 kHz and 3 MHz, respectively. The waveforms of the demodulated signals exhibit no significant noise or spurious oscillations, indicating that the measurements were performed under realistic conditions that are applicable to actual FM-AFM experiments.

Based on the amplitude and phase curves shown in Figure 5, we estimated the bandwidth and latency in the same way as we did for the PD–PC units. The results are summarized in Table 1 and Table 2. The latencies in the tables were estimated by performing curve fitting using Equation 1. These values are consistent with the delays visually estimated from the waveforms in Figure 6. Due to the existence of the phase feedback loop, the PLLs have narrower bandwidths and higher latencies than the corresponding PD–PC units. However, the following tendency is observable for both the PD–PC units and PLLs: Increasing *f*_0_ or using a HPF instead of a LPF improves the bandwidth and latency. The use of a HPF improves the bandwidth by 1.5–1.8 times for the PD–PC units and by up to 4.9 times for the PLLs, because the HPF not only suppresses the PD latency but also enables the use of the S-PLL design instead of the M-PLL design. Therefore, the difference between the latencies of the two PLLs is much more prominent than that between the two PD–PC units using HPF and LPF, respectively.

#### PLL with cantilever

**Figure 7:**
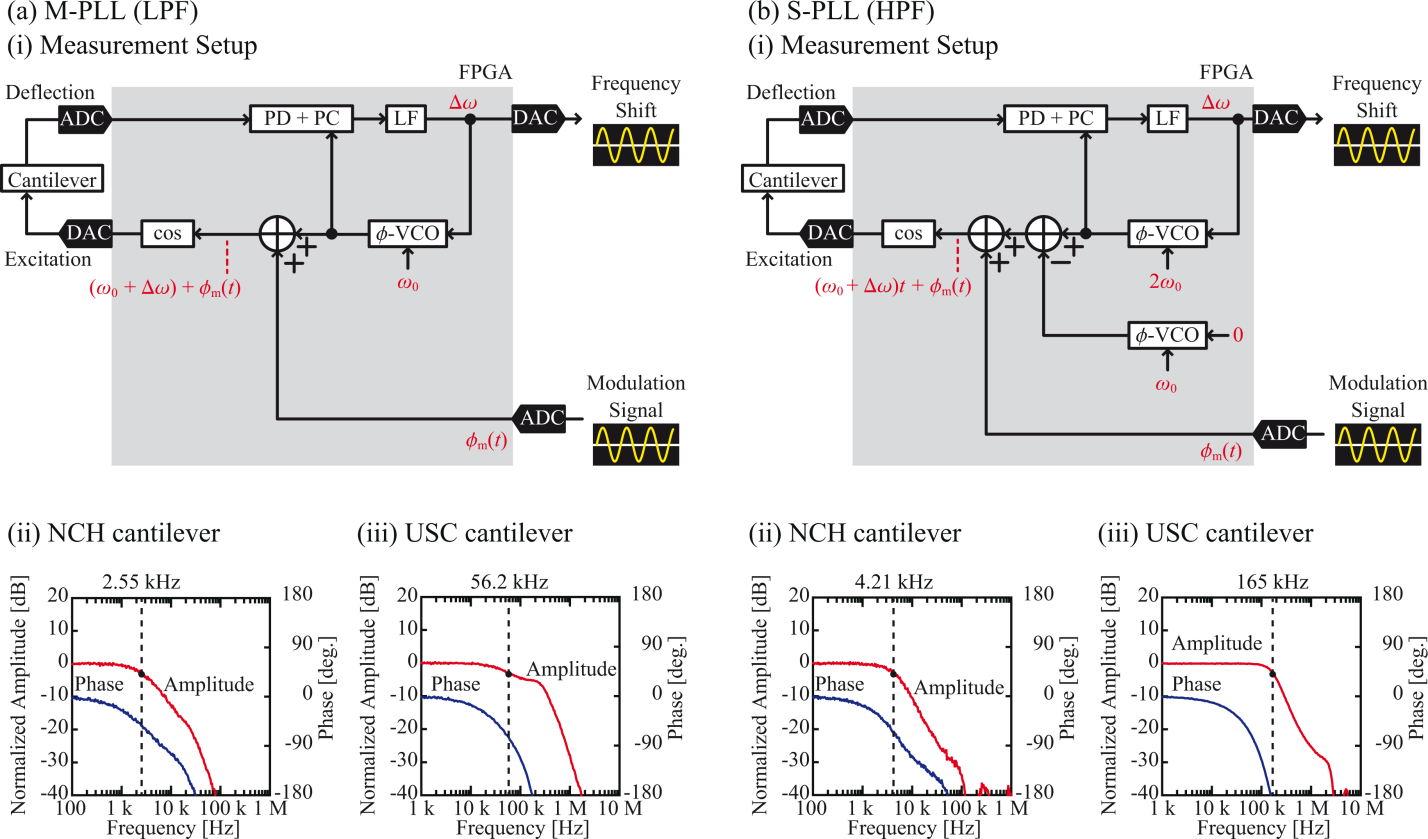
Performances of the (a) M-PLL with the LPF and (b) S-PLL with the HPF. (i) Block diagrams of the setups. (ii) and (iii) Frequency responses of the PLLs measured with the NCH (*f*_0_ = 151.46 kHz, *k* = 41.3 N/m and *A* = 0.1 nm) and USC (*f*_0_ = 3.44 MHz, *k* = 59.9 N/m and *A* = 0.1 nm) cantilevers, respectively.

To obtain the measurements described above, the cantilever excitation signal generated in the FPGA was directly fed into the PD–PC unit as a false cantilever deflection signal so that the frequency response of the PLL itself could be measured. However, in the actual AFM experiment, the excitation signal was output from the FPGA and returned to it through various components, such as the DAC, cantilever excitation unit, cantilever, cantilever deflection sensor, and ADC. Thus, the bandwidth and latency of the PLL should deteriorate when measured using a cantilever in an actual AFM setup. To investigate the influences of these components, we measured the amplitudes and phase responses of the PLLs by employing a cantilever immersed in a liquid using the setups illustrated in Figure 7(i). As standard and ultra-short cantilevers, we utilized the NCH and USC cantilevers, respectively, that were purchased from Nanoworld [27]. The LF gain was adjusted for each PLL in the manner described above.

Figure 7(ii) and Figure 7(iii) present the frequency responses of the PLLs measured with a cantilever in water. From these curves, we estimated the bandwidth and latency using the method described above. The results are summarized in Table 1 and Table 2. Comparison of these values with those obtained without a cantilever indicates that the bandwidth and latency deteriorated because of the inclusion of the cantilever. The higher *f*_0_ or use of the S-PLL instead of the M-PLL improves the bandwidth and latency. This tendency is the same as that observed in the measurements of the PD–PC and PLLs without cantilevers.

In Table 1 and Table 2, the ratios between the values measured using the LPF and HPF designs are also provided. The bandwidth improvement ratio decreases due to the inclusion of the cantilever. This reduction is much more significant for the NCH cantilever (from 4.87 to 1.65) than for the USC cantilever (from 4.87 to 2.94). For the NCH cantilever, the response time of the cantilever itself (1/*f*_0_ = 6.6 μs) is significant compared with the latency of the PD–PC unit (9.1 μs or 1.1 μs). Thus, its contribution reduces the difference between the performances of the two designs. In contrast, the response time of the USC cantilever (ca. 0.3 μs) is less than the latency of the PD–PC units, and its contribution to the performance is limited. Therefore, the difference between the performances of the two designs remains significant. In fact, comparison of the bandwidths of the PLLs with and without the cantilever indicates that the bandwidth reduction is much more significant for the NCH cantilever than for the USC cantilever.

**Figure 8:**
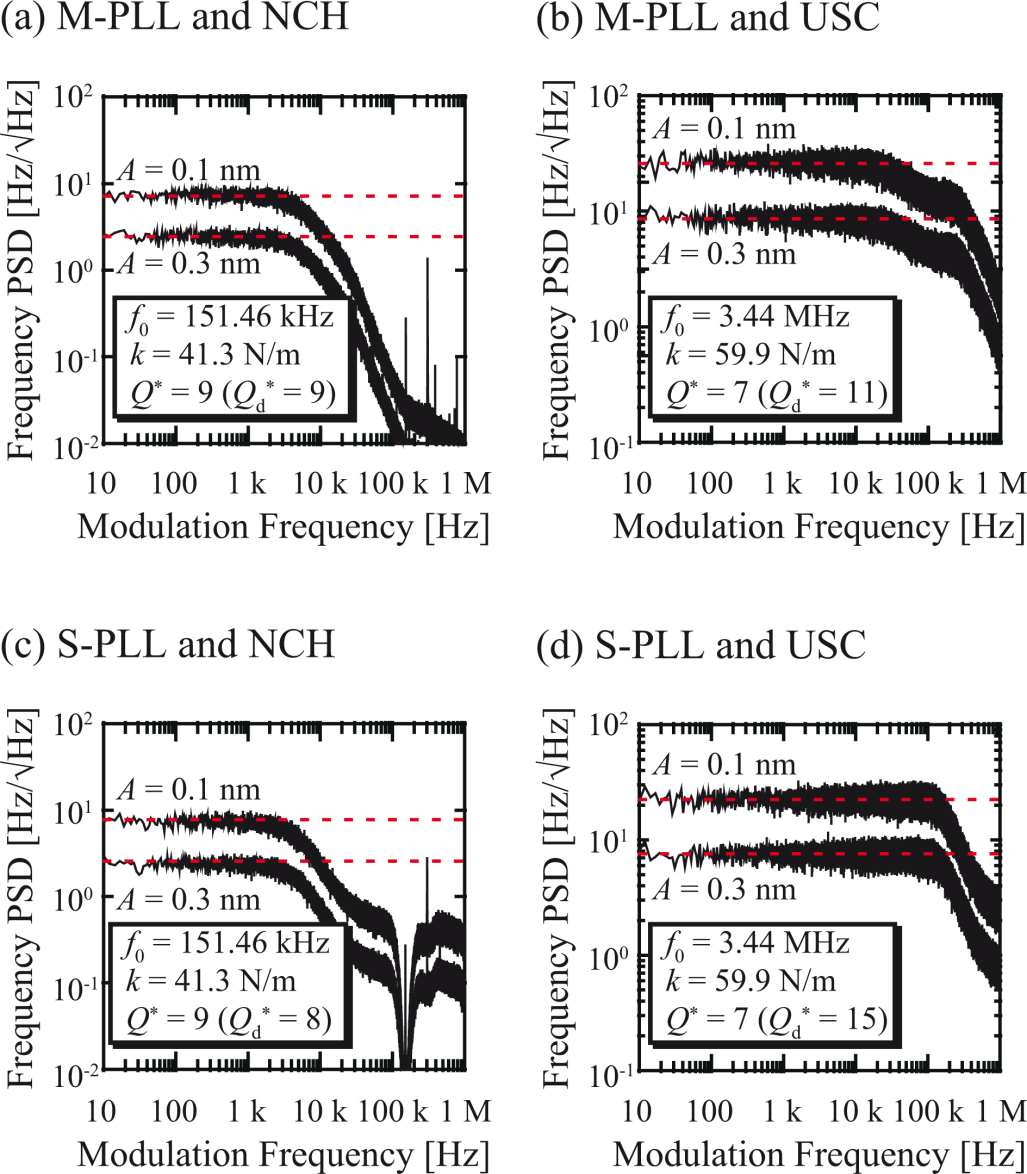
Frequency power spectral density (PSD) distributions of the output signals from the (a, b) M-PLL and (c, d) S-PLL measured with the cantilevers immersed in liquid and using the (a, c) NCH and (b, d) USC cantilevers.

Figure 8 shows the frequency spectral density distributions of the PLL output signals measured with the cantilevers immersed in liquid. In these measurements, we used the same cantilevers as in the amplitude and phase response measurements (Figure 7). In Figure 8, the solid black lines correspond to the measured raw data, while the red dotted lines indicate the noise originating from the cantilever thermal vibrations *n*_th_. These values were calculated using the following equation [1,33]:

[2]
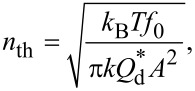


where *k*_B_, *T*, *k*, and *A* denote Boltzmann's constant, the absolute temperature, the cantilever spring constant, and the cantilever vibration amplitude, respectively. *Q*_d_* represents the apparent Q-factor in the liquid, which is calculated from the slope of the phase-versus-frequency curve [33]. The measured noise spectra are almost constant in the low-frequency range at values that agree with *n*_th_. This result demonstrates that the noise in the PLL output signals mostly originates from the cantilever thermal vibration and that the noise generated inside the PLLs is negligible. The cut-off frequencies of the measured spectra correspond to the bandwidths listed in Table 1 and Table 2, as expected from the basic PLL principle. Overall, these results confirm that the developed PLLs can provide the optimal noise performance in FM-AFM experiments performed in liquids for both the NCH and USC cantilevers.

#### High-speed FM-AFM imaging with true atomic resolution

**Figure 9:**
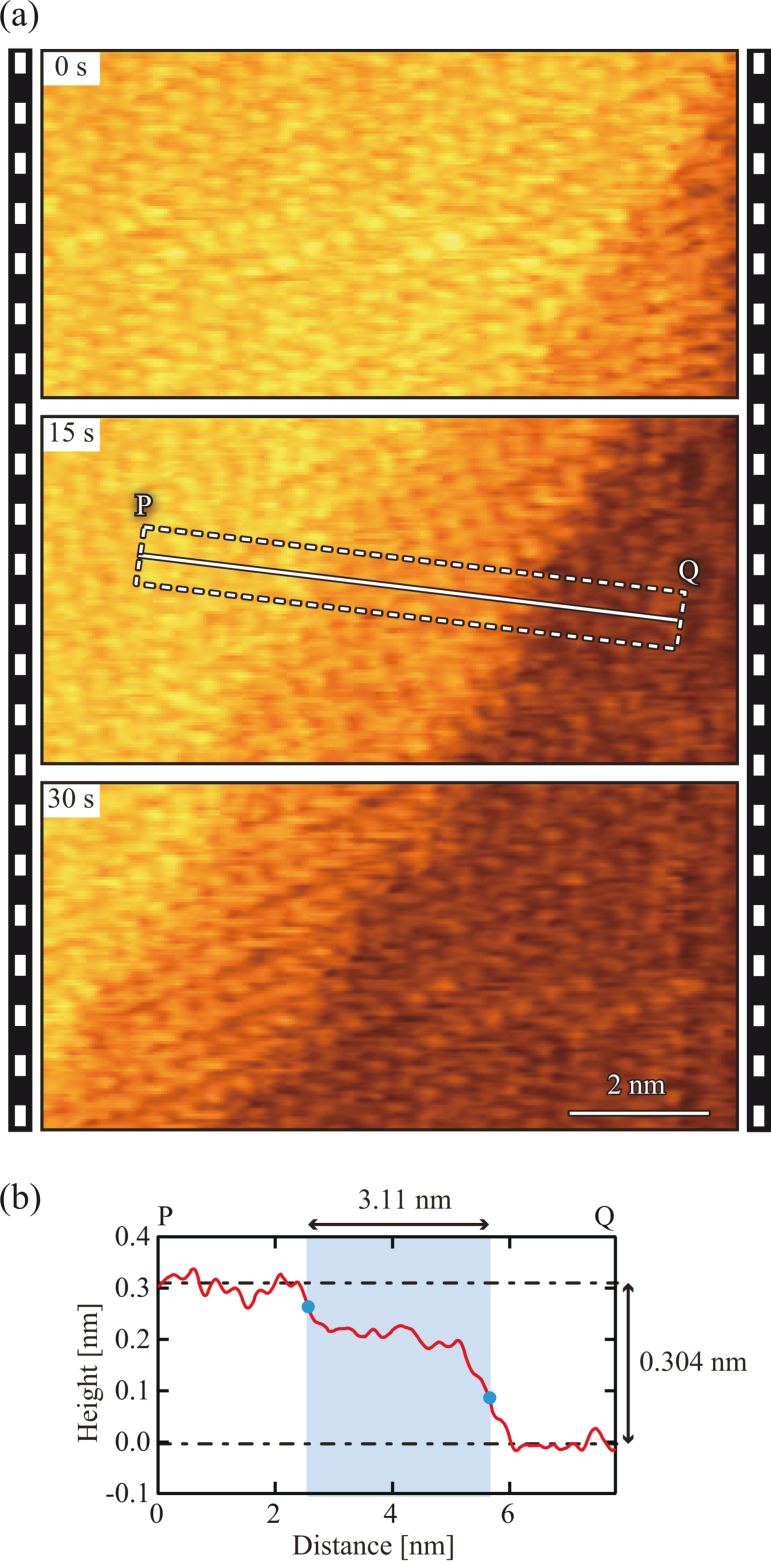
(a) High-speed FM-AFM images of calcite (10−14) surface obtained in water at 0.5 s/frame (10 × 5 nm^2^, 500 × 250 pix^2^). (b) Height profile measured along line PQ in panel (a). The profile was averaged over the width indicated by the dotted lines.

To investigate the applicability of the developed S-PLL to high-speed atomic-resolution imaging in a liquid, we imaged calcite dissolution in water. Figure 9a provides three representative frames selected from many successive FM-AFM images (see Supporting Information File 1 for a movie consisting of all the successive images), which was obtained at 0.5 s/frame. These AFM images depict the movement of the atomistic step from the lower right to the upper left due to the dissolution at the step edge. In spite of the high imaging speed, atomic-scale contrast is clearly evident even near the step edge, demonstrating that the developed S-PLL is capable of high-speed and true atomic-resolution imaging in liquids at an imaging speed faster than 1 s/frame.

Figure 9b shows the averaged height profile measured along line PQ in Figure 9a(ii). This profile reveals that the difference between the height of the upper and lower terraces is ≈0.3 nm. This value corresponds to the height of a single atomistic step on the calcite (10-14) surface (0.304 nm). The AFM images also indicate the existence of a transition region having a width of 2–3 nm and a height of ≈0.2 nm between the upper and lower terraces. As we previously reported [26], this region is not a tip artefact, but rather an intermediate state formed during calcite dissolution. In the previous report [26], we discussed the origin of this region in detail. By comparing the AFM images with the molecular dynamics simulation results, we proposed that the transition region is most likely to be a Ca(OH)_2_ layer. In this study, this transition region was newly observed by direct imaging using high-speed FM-AFM. This result demonstrates the effectiveness of the developed S-PLL in practical high-speed FM-AFM experiments.

## Conclusion

In this study, we compared different PLL designs for high-speed FM-AFM in detail. Specifically, we compared the design concepts of the conventional PLL using a LPF and the newly proposed PLL using a HPF. In the standard LPF design, a LPF with high latency is located inside the phase feedback loop, causing the bandwidth to be relatively narrow. In the proposed HPF design, a HPF with low latency is employed for phase detection and is located outside the phase feedback loop. This design enables the use of a subtraction-based phase comparator in the phase feedback loop, providing a relatively wide bandwidth. We implemented these two types of PLLs in the same FPGA chip and quantitatively compared their performances. The results revealed that the HPF design provides a bandwidth three times wider (165 kHz) than that of the LPF design when it is used with an ultra-short cantilever in a liquid (*f*_0_ = 3.44 MHz). With the developed wideband PLL, we performed high-speed FM-AFM imaging of calcite dissolution at 0.5 s/frame in water and directly visualized atomic-scale dynamic events near the step edges. These results demonstrate the effectiveness of the proposed PLL design for high-speed FM-AFM.

## Supporting Information

Imaging speed: 0.5 s/frame, 10 × 5 nm^2^, 500 × 250 pix^2^. Three images were selected and are shown in Figure 9a.

File 1 Successive FM-AFM images of calcite surface obtained in water.
